# Challenging the One-hour Sepsis Bundle

**DOI:** 10.5811/westjem.2018.11.39290

**Published:** 2019-02-04

**Authors:** Annahieta Kalantari, Salim R. Rezaie

**Affiliations:** * Penn State Health Milton S. Hershey Medical Center, Department of Emergency Medicine, Hershey, Pennsylvania; † Greater San Antonio Emergency Physicians, Department of Emergency Medicine, San Antonio, Texas; ‡ Greater San Antonio Emergency Physicians, Department of Internal Medicine, San Antonio, Texas

## Abstract

In April 2018, the Surviving Sepsis Campaign (SSC) released an updated sepsis bundle, which combines directives previously listed in the three-hour and six-hour bundles. The authors discussed the reasoning and evidence supporting these changes. However, there are data that suggest these recommendations may be contrary to the best available evidence. Our purpose here is to highlight the areas where evidence is only as strong as the methodological constructs of the research used. This article is a narrative review of the available, limited evidence on which the one-hour bundle was based.

## INTRODUCTION

In April 2018, the Surviving Sepsis Campaign (SSC) released an updated sepsis bundle (Table 1), which combines directives previously listed in the three-hour and six-hour bundles. In this update the authors noted that *“*when they [the bundles] were introduced, the bundle elements were designed to be updated as indicated by new evidence and have evolved accordingly.”^1^ Yet, some of the studies included in these recommendations are of poor quality and have methodological issues, making it dangerous to draw dogmatic conclusions about generalizability to all septic patients. Additionally, the one-hour bundle makes recommendations that are still shrouded in unresolved controversies. Furthermore, the exact sepsis definitions used within the article are nebulous, and the definition of time zero (i.e., at triage) may not allow successful implementation of the bundle. The one-hour bundle may have a bigger implication with regard to future hospital reimbursements and, most importantly, patient care. This article addresses these challenges and a few others in greater detail.

### Challenge 1: Definition of Sepsis

Before discussing the individual elements of the bundle, we must first address the fact that there is no single, clear definition of sepsis currently being used to screen for these patients. Clinicians practicing in the United States have three options from which to choose when defining patients presenting with a sepsis spectral illness: the Sepsis 2.0 definitions, the Centers for Medicare and Medicaid Services (CMS) definitions, or the Sepsis 3.0 definitions. Each is listed in Table 2.

The 2018 SCC one-hour bundle paper refers to the 2016 SSC guidelines “for further discussion and evidence related to each element and to sepsis management as a whole.” Does this mean we should refer to the 2016 guidelines regarding sepsis definitions? If we do, there are no clinical parameters within this document. With regard to verbal definitions, the 2016 SSC iteration accepted some of the Sepsis-3.0 proposals and eliminated severe sepsis as a category. The SSC also accepted the proposed verbal definitions for sepsis and septic shock. However, qSOFA (quick sequential organ failure assessment) was not accepted or recommended as best practice, and systematic inflammatory response (SIRS) along with all other specific clinical parameters of end organ dysfunction were eliminated from the recommendations.^6^

There are no defined elements of sepsis offered to clinicians in order to determine which patient population requires application of the one-hour bundles. Are we using Sepsis 2.0, Sepsis 3.0, or the CMS definitions? If it is Sepsis 3.0, the sensitivity of qSOFA is too low for emergency department (ED) application and patients will be missed.^7^–^16^ Additionally, multiple national organizations have not accepted the Sepsis 3.0 definitions. There is no gold standard definition established to trigger any resuscitative cascade.^17^ The exact definitions with corresponding clinical parameters must be clearly defined in the 2018 recommendations, and they must be evidence based.

### Challenge 2: Bundle Compliance and Protocolized Sepsis Care

The authors of the one-hour bundle state, “The compelling nature of the evidence in the literature … has demonstrated an association between compliance with bundles and improved survival in patients with sepsis and septic shock ….*”*^1^ Patients with sepsis and those with septic shock are two very different patient populations. The SSC one-hour bundle paper cites a retrospective review by Seymour et al. that demonstrated improved mortality outcomes in patients with septic shock who received the three-hour bundle. There was no survival benefit in patients who were not in septic shock.^18^ This evidence does not support the application of these bundles to patients with sepsis. With regard to patients with septic shock, three large, randomized control trials – ARISE, ProMISe and ProCESS – all demonstrated no significant difference in patient mortalities who were treated via usual care vs protocols.^19^–^21^ There are no definitive data to support that bundle compliance improves mortalities in septic patients, and the data are mixed regarding improved survival in patients with septic shock.

### Challenge 3: Time Zero and Emergency Medicine

The 2018 SSC bundle states, “Consistent with previous iterations of the SSC sepsis bundles, ‘time zero’ or ‘time of presentation’ is defined as the time of triage in the ED or if referred from another location, from the earliest chart annotation consistent with all elements of sepsis (formerly severe sepsis) or septic shock ascertained through chart review*.”*^1^ Up to 53% of patients will not demonstrate evidence of severe sepsis or septic shock at time of triage.^22^ In the SSC one-hour bundle paper, authors compared the care of patients presenting with polytrauma, acute myocardial infarction (MI) and cerebrovascular accident to those presenting with sepsis. Unlike sepsis, these other conditions have very distinct pathophysiologic causes, consistent clinical effects and rapid screening processes.

Sepsis presentations are dependent on causative organisms, patient comorbidities and other confounding factors. Many times there is no indication that patients are severely ill upon initial evaluation. Some data collected in laboratory tests suggested a higher degree of illness, but these values rarely are resulted rapidly enough to identify and initiate treatment within one hour of patient arrival. Traumas, MIs, and strokes do not require laboratory values for screening and identification.

Because the definitions are not identified, it is unclear which patients require rapid assessment at time zero. Many patients present to the ED with SIRS criteria, which can be due to a variety of conditions other than infection and sepsis. The differential diagnosis of a tachycardic patient presenting with abdominal pain encompasses a nonemergent diagnosis of pain from gastritis all the way to impending septic shock due to a perforated viscous. Very few EDs have the capability to make the exact diagnosis and initiate resuscitative efforts from triage. Unless the patient presents with other signs and symptoms suggesting a more emergent diagnosis, treatment will begin later than one hour after triage.

The one-hour bundle challenges providers to send nearly every SIRS-positive patient through a rapid sepsis screening process, which is not feasible or compatible within the daily operations of the ED.^23^ Time zero should not be time of triage. It should be time of physician suspicion of infection.

Finally, while all the authors of the one-hour SSC bundle are well-respected intensivists, unfortunately they are unfamiliar with the challenges of the ED. For most patients, this first hour of resuscitation will occur in the ED. Inclusion of an emergency physician, who has knowledge and experience of ED operations, would allow for better collaboration and success in implementation of care bundles and for exclusion of recommendations that may not be feasible to implement in the ED and may also cause harm.^24^

### Challenge 4: Lactate

The authors state there is “low quality of evidence*”* for initial measurement of lactate with repeat measurements for lactate >2 millimoles per liter (mmol/L).^1^ While there is evidence that elevated lactates are associated with an increased mortality and lactate clearance is associated with lower mortality,^25^–^27^ the exact lactate level that should trigger aggressive resuscitative effort remains unknown. Traditionally, most studies used a lactate of greater than 4 mmol/L.^19^–^21^,^25^,^28^ Since 2005, researchers have studied varying lactate levels and associated mortality rates.

Shapiro and colleagues performed a prospective cohort study demonstrating a 4.9% mortality for patients with an initial lactate of 0–2.4 mmol/L, 9.0% mortality for patients with initial lactates between 2.5 and 3.9 mmol/L and a 28.4% mortality for patients with an initial lactate >4 mmol/L.^29^ In 2009, Mikkelsen et al. risk-stratified patient mortality according to varying lactate levels and found patients without evidence of shock had an 8.7% mortality rate with lactate levels <2 mmol/L, a 16.4% mortality rate with lactate levels 2–3.9 mmol/L and 31.8% with lactate levels >4 mmol/L. In patients with shock, corresponding mortality rates were 15.4%, 37.3% and 46.9%.^30^ In 2015, Bhat et al. conducted a retrospective review that revealed 28-day mortalities were 12.7% for patients with an initial lactate <2 mmol/L, 19.5% for patients with an initial lactate between 2.0 and 4 mmol/L and 24.6% for those with lactates >4.0 mmol/L.^26^ None of the studies demonstrated a consistent, clear delineation in which an intermediate lactate level was associated with a sudden increase in mortality,^26^,^29^,^30^ yet we are provided with the cut-off value of 2 mmol/L.

### Challenge 5: Fluids

The authors state there is “low quality of evidence*”* for the administration of 30 milliliters per kilogram (ml/kg) of crystalloid fluids.^1^ With regard to fluid resuscitation, multiple studies have demonstrated aggressive fluid resuscitation and positive fluid balances are harmful and increase mortality.^31^–^36^ In the Seymour et al. study discussed above, there was no association between improved survival rates and fluid administration.^18^ Yet the fluid component has been moved to begin within one hour. Additionally, the exact quantity of fluid that defines a fluid bolus varies in different studies.^19^–^21^,^37^–^39^ A prescriptive fluid bolus amount that does not consider individual patient needs and comorbidities is potentially deleterious. Clinicians should have the opportunity to judge and determine the amount of fluids that his/her patient requires.

### Challenge 6: Timing of Antibiotics

In 2006, Kumar et al. published results from a retrospective study demonstrating an average increase in mortality by 7.6% for every one-hour delay in the administration of antibiotics in patients presenting with septic shock.^40^ These data were incorporated into the 2008 SSC guidelines^41^ and extrapolated to the treatment of patients presenting with severe sepsis as well, even though this was not the patient population studied in Kumar’s paper. Several follow-up studies were performed to evaluate associations between mortality and timing of antibiotic administration. A cohort analysis from the EMSHOCKNET study found no association between in-hospital mortality and the time from ED triage to administration of antibiotics during the first six hours of resuscitation, but did find an increased risk of death if antibiotics were delayed until after the recognition of shock.^42^

In a 2015 systemic review and meta-analysis, authors demonstrated no significant survival benefit of administering antibiotics within three hours of ED triage or within one hour of septic shock recognition in severe sepsis and septic shock.^43^ Seymour et al. demonstrated improved survival rates in patients receiving antibiotics within three hours, but they did not extend this to within one hour and noted that the improved survival rates appeared to be stronger among patients receiving vasopressors than among those who were not.^18^ Most recently, the PHANTASi (Prehospital ANTbiotics Against Sepsis) trial demonstrated no differences in 28-day or 90-day mortality between sepsis, severe sepsis or septic shock patients receiving antibiotics in the ambulance en route to the hospital vs those patients who received usual care and were administered antibiotics after arrival to the hospital.^44^

Lastly, analysis of the SSC registry demonstrated that approximately one-third of septic shock patients do not receive broad-spectrum antibiotics within three hours of ED presentation,^45^ yet the time window was decreased to one hour. The evidence does not support this strict timeline on antibiotic administration to all septic patients. Additionally, antibiotics are not without harm. Increased use contributes to increased microbial resistance, the potential to increase *Clostridium difficile* colitis, as well as other adverse events. Administration of antibiotics to meet a timeline that is not evidence based will result in an increase of inappropriate antibiotic use.

### What Does This All Mean?

As history has a way of repeating itself, it is highly likely that this proposed one-hour bundle will be used as a marker of quality by CMS. The downstream effects of this decision will result in hospital reimbursement cuts in an already fiscally-narrow existence. Additionally, once these measures are required for reimbursement, hospital administrators will pressure clinicians to meet these broadly applied, checked items. This has several implications and the potential for deleterious outcomes.

As discussed above, up to 53% of patients diagnosed with severe sepsis and septic shock do not present with evidence of such in triage. As it takes time to evaluate these patients, make a diagnosis and initiate treatment, many will not meet initiation of the one-hour bundle in time. In an effort to meet the bundle, patients will receive antibiotics unnecessarily or will receive inappropriate antibiotics because the diagnosis has yet to be made in a setting where the risk does not outweigh the benefit. Some patients will receive intravenous fluids in amounts that are harmful, resulting in higher morbidities and mortalities.

Forcing a physician to practice recommendations that are not backed by high-quality evidence will unnecessarily harm patients and put the very people we are to care for at high risk of poor outcomes. In its current form, the one-hour bundle faces many challenges and requires several revisions. This bundle should be revised to state: “*We suggest that these bundles should be initiated within one hour of physician suspicion of infection causing hypotension or lactate greater than 4 mmol/L. A fluid bolus of 30ml/kg should be administered to patients when it is safe to administer such a volume*.” Until this bundle is updated to include this statement, it is not appropriate or ready for bedside application in the ED setting. We, practicing emergency physicians, should have the ability to choose the components that are applicable to our patients.

## Figures and Tables

**Table 1:** Surviving Sepsis Campaign one-hour bundle.

Bundle element	Grade of recommendation and level of evidence
Measure lactate. Re-measure if initial lactate > 2 mmol/L.	Weak recommendation. Low quality of evidence.
Obtain blood cultures prior to administration of antibiotics.	Best practice statement.
Administer broad-spectrum antibiotics.	Strong recommendation. Moderate quality of evidence.
Rapidly administer 30 ml/kg crystalloid for hypotension or lactate ≥4 mmol/L.	Strong recommendation. Low quality of evidence.
Apply vasopressors if patient is hypotensive during or after fluid resuscitation to maintain MAP ≥ 65 mm Hg.	Strong recommendation. Moderate quality of evidence.

*mmol/L*, millimoles per liter; *ml/kg*, milliliters per kilogram; *mmHg*, millimeters of mercury; *MAP*, mean arterial pressure.

**Table 2:** Various definitions for sepsis spectral illnesses.

	Sepsis 2.0^2^,^3^	CMS^4^	Sepsis-3.0^5^	2016 SCC Guidelines^6^
SIRS	Temperature > 38°C or < 36°C Heart rate > 90 bpm Respiratory rate > 20 or PaCO_2_ < 32 mmHg White blood cell count > 12,000/cu mm, < 4,000/cu mm or > 10% bands	No change	Eliminated. qSOFA introduced Respiratory rate > 22 Altered mental status Systolic blood pressure < 90 mmHg	No SIRS. No qSOFA.
Sepsis	Infection and two or more SIRS	No change	Infection and two qSOFA criteria	Infection and end organ dysfunction. No clinical criteria offered.
Severe Sepsis	Sepsis and end organ dysfunction defined as: Sepsis-induced hypotension Lactate above upper limits of laboratory normal Urine output < 0.5 ml/kg/hr × two hours PaO_2_/FiO_2_< 250 in absence of pneumonia PaO_2_/FiO_2_< 200 in presence of pneumonia Creatinine > 2.0 mg/dL Bilirubin > 2 mg/dL Platelet count < 100,000/uL INR > 1.5	Sepsis and end organ dysfunction. Lactate > 2	Eliminated	Eliminated
Septic Shock	Sepsis and a SBP < 90 mmHg or a reduction of 40 mm Hg from baseline or evidence of low perfusion after adequate fluid bolus.	Initial lactate > 4 or SBP < 90 mm Hg after 30 mL/kg fluid bolus	SBP < 90 mmHg AND lactate > 2 after adequate fluid resuscitation	Subset of sepsis with circulatory and cellular/metabolic dysfunction associated with a higher risk of mortality. No clinical criteria offered

*SIRS*, systemic inflammatory response syndrome; *CMS*, Centers for Medicare and Medicaid Services; *SCC*, Surviving Sepsis Campaign; *bpm*, beats per minute; *cu mm*, cubic millimeter; *qSOFA*, quick sequential organ failure assessment; *ml/kg/hr*, milliliter per kilogram per hour; *PaO_2_*, partial pressure of oxygen; *FiO_2_*, fraction of inspired oxygen; *INR*, international normalized ratio; *mg/dL*, milligram per deciliter; *MAP*, mean arterial pressure; *SBP*, systolic blood pressure.

* All lactate levels in millimoles per liter values.
